# Estimation of 24-Hour Intraocular Pressure Peak Timing and Variation Using a Contact Lens Sensor

**DOI:** 10.1371/journal.pone.0129529

**Published:** 2015-06-15

**Authors:** John H. K. Liu, Kaweh Mansouri, Robert N. Weinreb

**Affiliations:** 1 Hamilton Glaucoma Center, Department of Ophthalmology, University of California San Diego, La Jolla, California, United States of America; 2 Glaucoma Sector, Division of Ophthalmology, Geneva University Hospitals, Geneva, Switzerland; University of Iowa, UNITED STATES

## Abstract

**Purpose:**

To compare estimates of 24-hour intraocular pressure (IOP) peak timing and variation obtained using a contact lens sensor (CLS) and using a pneumatonometer.

**Methods:**

Laboratory data collected from 30 healthy volunteers (ages, 20-66 years) in a randomized, controlled clinical trial were analyzed. Participants were housed for 24 hours in a sleep laboratory. One randomly selected right or left eye was fitted with a CLS that monitored circumferential curvature in the corneoscleral region related to the change of IOP. Electronic output signals of 30 seconds were averaged and recorded every 5 minutes. In the contralateral eye, habitual IOP measurements were taken using a pneumatonometer once every two hours. Simulated 24-hour rhythms in both eyes were determined by cosinor fitting. Simulated peak timings (acrophases) and simulated data variations (amplitudes) were compared between the paired eyes.

**Results:**

Bilateral change patterns of average 24-hour data for the group were in parallel. The simulated peak timing in the CLS fitted eye occurred at 4:44 AM ± 210 min (mean ± SD) and the IOP peak timing in the contralateral eye at 4:11 AM ± 120 min (P=0.256, Wilcoxon signed-rank test). There was no significant correlation between the simulated data variations in the paired eyes (P=0.820, linear regression).

**Conclusions:**

The 24-hour CLS data showed a simulated peak timing close to the 24-hour IOP peak timing obtained using the pneumatonometer. However, the simulated variations of 24-hour data in the paired eyes were not correlated. Estimated 24-hour IOP rhythms using the two devices should not be considered interchangeable.

## Introduction

Measurements of intraocular pressure (IOP) outside office hours can provide useful information for glaucoma diagnosis and treatment [1]-[6]. Examination of IOP during a 24-hour period is usually performed with subjects housed in a sleep laboratory or an inpatient facility [7]-[9]. For practical reasons, data are typically collected once every few hours and there is a need of awakening subjects for tonometry during the sleep period. Recently, a wireless contact lens sensor (CLS, Triggerfish; Sensimed, Lausanne, Switzerland) has been developed for monitoring 24-hour IOP at home without the need of awakening subjects during sleep [10], [11]. Several reports have demonstrated good tolerability of the CLS for 24-hour recordings, but the clinical applications of the CLS need to be investigated [12]-[19]. By design, the CLS detects the circumferential change in the corneoscleral curvature that is under the influence of IOP. Output signals from the CLS are in electronic units of millivolt (mV). How to convert the CLS signals to accurate IOP values is currently unknown, but a unique 24-hour CLS signal pattern is readily available for each test individual.

We and others have studied 24-hour IOP patterns for various groups of experimental subjects in a sleep laboratory [20]-[26]. Mathematically simulated individual 24-hour IOP rhythms often showed their peak timings outside office hours. Assuming that the CLS signal change is closely related to the IOP change [11], the 24-hour CLS output signals should also provide information of the 24-hour IOP rhythm commonly represented by the simulated peak timing and the simulated data variation [27]. Therefore, it is important to examine whether or not the peak timing and variation of the 24-hour CLS data are in agreement with the parameters of 24-hour IOP rhythm determined by an established method. In the present study, we compared the mathematically simulated peak timing and variation of 24-hour CLS output signals in one eye with the simulated 24-hour IOP rhythm in the contralateral eye in a group of healthy adults. In healthy adults, 24-hour IOP rhythms in the paired eyes are relatively symmetrical [28].

## Methods

Data gathered in a prospective clinical trial for the safety and effectiveness of Triggerfish CLS were used. This clinical trial (http://www.clinicaltrials.gov; NCT01390779) adhered to the tenets of the Declaration of Helsinki, in accordance with the Health Insurance Portability and Accountability Act, and was approved by the Institutional Review Board of the University of California, San Diego. Written informed consent was obtained from each participant after an explanation of the study including the potential side effects.

All subjects underwent a complete ophthalmic examination consisting of a medical history, best-corrected visual acuity, refraction, ocular biometry, central corneal thickness using an ultrasonic pachymeter (Pachette 2; DGH Technology, Exton, PA), slit-lamp biomicroscopy, dilated ophthalmoscopy, and Goldmann applanation tonometry during office hours. Exclusion criteria included the presence of ocular disease other than primary open-angle glaucoma, a spherical equivalent more than 4 diopters, a cylinder equivalent more than 2 diopters, corneal or conjunctival abnormalities hindering adaptation of silicone contact lens, and IOP asymmetry of more than 3 mmHg.

Enrolled subjects were selected based on having a regular daily sleep of approximately 8 hours. Before the laboratory experiment, they were instructed to maintain an accustomed 8-hour sleep for 7 days. Subjects were given a wrist-mounted device (Actiwatch; Mini Mitter, Sunriver, OR) and a wake/sleep log to keep track of physical activity and light exposure. They were also asked to abstain from alcohol for 3 days and regular coffee for 1 day. Subjects reported to the university eye clinic before 2 PM. An envelope with a randomized assignment of right eye or left eye by computer was opened. Measurements of central corneal thickness were taken from both eyes and CLS was fitted in the assigned eye [27]. When the CLS recorder was connected, the device software averaged the first 30-sec CLS output signals (10 Hz) and stored the average as an arbitrary reference of 0 mV. Averaging 30-sec CLS output signals had been programmed to continue every 5 minutes and the mV difference from the arbitrary reference was recorded. A 24-hour CLS recording may contain 288 data points overall.

Subjects with a successful CLS fitting and data recording moved to the adjacent sleep laboratory at approximately 2 PM. Laboratory conditions were controlled as described previously [26]. The 8-hour period of darkness in each sleep room was adjusted close to the individual’s sleep cycle, and times for the IOP measurements in the contralateral eye were individualized accordingly. Clock times for lights off (between 10 PM and 11:30 PM) were normalized for data presentation as if each subject slept from 11 PM to 7 AM. Subjects were provided with a diary for the recording of physical activities, intake of meals, and other events every half hour.

Measurements of habitual IOP in the contralateral eye were taken once every 2 hours by experienced technicians using a pneumatonometer (Model 30 Classic; Reichert, Depew, NY). Measurements were taken from subjects sitting during the diurnal/wake period and supine during the nocturnal/sleep period. Proparacaine 0.5% was applied for local anesthesia. A hard-copy printout was inspected visually [21]. Before the nocturnal/sleep period, IOP was measured at 3:30 PM, 5:30 PM, 7:30 PM, and 9:30 PM. Subjects were instructed to sit for 5 minutes. Blood pressure and heart rate were measured immediately before the IOP measurements. Lights were turned off at 11:00 PM and nocturnal measurements were taken at 11:30 PM, 1:30 AM, 3:30 AM, and 5:30 AM. Subjects were awakened, if necessary, and the measurements of blood pressure, heart rate, and IOP were taken supine under dim light (<10 lux). Light exposure was kept to a minimum during the nocturnal period. When the sleep period ended at 7 AM, room lights were turned on and subjects were awakened, if necessary. Diurnal measurements were taken again at 7:30 AM, 9:30 AM, 11:30 AM, and 1:30 PM in the laboratory as described previously. Food and water were available at all times. Meal times and indoor activities were not regulated. Between the scheduled IOP measurements using the pneumatonometer every two hours, subjects’ ambulatory postures were not regulated.

The CLS recorder automatically stopped data collection after 24 hours. The CLS was removed from the subject in the eye clinic, the activity diary was collected, and an ophthalmologic examination including the measurement of central corneal thickness was performed. Data were downloaded from the CLS recorder to a computer via Bluetooth. This information was decoded by the CLS manufacturer and all raw data were available to the investigators for analyses. For the registered clinical trial, there were two outcome measures a priori: the wake/sleep slope of CLS output signals versus IOP and the nocturnal CLS pulse frequency related to the subject’s heart beats. Evaluations of these outcome measures and adverse events during the trial are reported separately from the present study [29]. The clinical trial had enrolled 31 healthy individuals and 2 patients with primary open-angle glaucoma. Since 24-hour IOP rhythms in the paired eyes of glaucoma patients can be relatively asymmetric judging by their peak timings and data variations [28], only data from the healthy individuals were included in the present study. We evaluated the 24-hour rhythms in the paired eyes considering all the enrolled healthy subjects including one subject whose data were not qualified for the preset outcome measures due to a small wake/sleep IOP slope.

Two sets of laboratory data were used; recorded CLS output signals (30-sec averages) every 5 minutes in one eye and IOP data from the contralateral eye every 2 hours. Using the least squares cosinor method [27], [30], the 24-hour rhythm in each of the paired eyes was estimated. The cosinor method presented the parameters of mesor, acrophase, and amplitude. The mesor represented 24-hour data average. The acrophase represented the phase timing (simulated peak timing). The amplitude (half distance between the cosinor-fit maximum and minimum) provided simulated 24-hour data variation. A null hypothesis that group phase timings were distributed randomly in 24 hours was tested using the Rayleigh test [31]. A rejection of the null hypothesis would indicate a synchronized 24-hour phase timing for the group.

In the eye fitted with CLS, 24-hour data average (mesor) was based on an arbitrary reference during the initial stage of contact lens wearing (average of the first 30-sec data), which may not provide useful comparison to the 24-hour IOP average in the contralateral eye. The simulated peak timing of 24-hour CLS data and the simulated peak timing of 24-hour IOP data in the contralateral eye were compared using the non-parametric Wilcoxon signed-rank test. P < 0.05 was considered as statistically significant. Since CLS provided output signals in mV and pneumatonometer provided IOP data in mmHg and the fact that a conversion formula is unknown, a direct comparison between the paired amplitudes of the simulated 24-hour rhythms was difficult. Instead, a linear regression analysis was performed to evaluate the correlation between the two amplitudes.

We recognized that, when IOP related data were collected in the paired eyes by different methods, more factors can influence the estimations of 24-hour rhythm compared to the use of a single method. In order to evaluate the clinical significance of the impact from the use of two methods, the time interval between the paired peak timings of 24-hour rhythm was compared to the related time interval when bilateral IOP data were collected using only the pneumatonometer. For each subject in the present study, the absolute difference in the peak timings of 24-hour CLS data and the contralateral 24-hour pneumatonometer data, regardless of the order of appearance, was calculated. For bilateral IOP data collected using only the pneumatonometer under similar laboratory conditions, the absolute time intervals between the peak timings had been calculated in a recent report including 91 healthy subjects [28]. The comparison of absolute time intervals between the paired peak timings for subjects in the present study (CLS/pneumatonometer) and the absolute time intervals in that recent report (pneumatonometer/pneumatonometer) was performed using the non-parametric Mann-Whitney rank-sum test.

## Results

All 31 healthy subjects had completed the experimental procedure. However, continuous CLS data could not be retrieved from one subject and this subject was excluded from data analyses. The remaining 30 subjects included 23 whites, 6 Asians, and 1 black. There were 16 males and 14 females. They had an age of 34.4 ± 13.4 (mean ± SD; ages 20–66 years), height of 171.2 ± 10.4 cm, weight of 73.9 ± 16.4 kg, and body mass index of 25.0 ± 4.1 kg/m^2^. The participants had one eye fitted with CLS and the contralateral IOP measured using a pneumatonometer. Comparing the two sets of eyes, there was no difference in Goldmann IOP, axial length, refractive state, and central corneal thickness based on the enrollment data (Table 1).

**Table 1:** Characteristics of the paired study eyes.

	Eye with CLS^a^	Contralateral eye	Difference^b^
Goldmann IOP (mmHg)	17.7 ± 2.7	17.8 ± 3.0	0.0 ± 1.5
Axial length (mm)	23.77 ± 0.90	23.76 ± 0.88	0.01 ± 0.22
Refraction (diopter)	-0.63 ±1.18	-0.60 ±1.19	-0.02 ± 0.32
Central corneal thickness (μm)	566.4 ± 34.6	566.3 ± 35.7	0.1 ± 7.6

^a^CLS, contact lens sensor.

^b^None of the difference is statistically significant using the paired t-test (N = 30).

**Fig 1:**
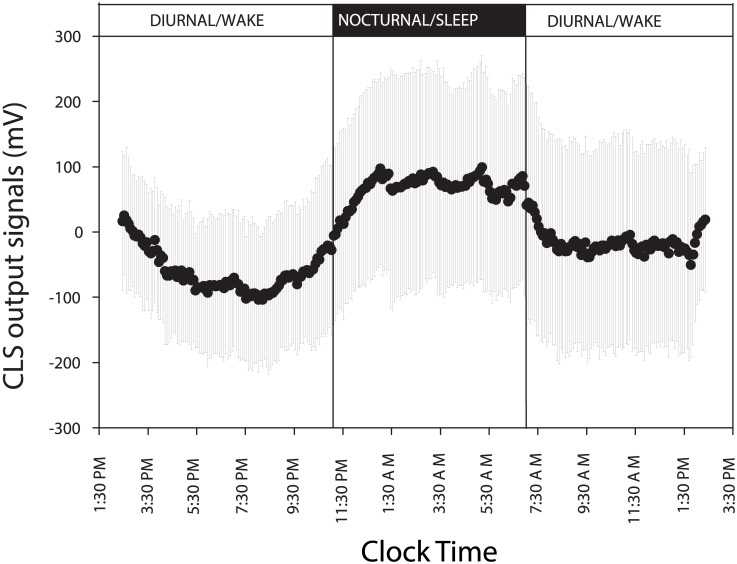
Twenty-four-hour pattern of contact lens sensor (CLS) output signals in ambulatory healthy adults. N = 30. Error bars, SD.

**Fig 2:**
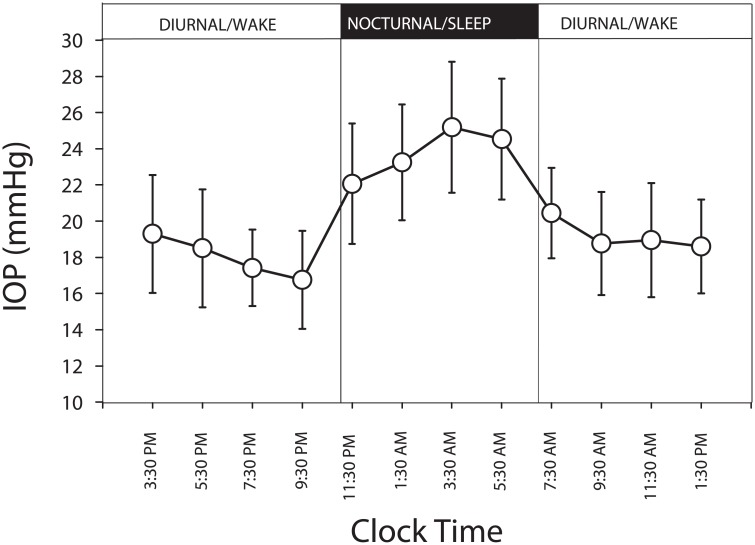
Twenty-four-hour intraocular pressure (IOP) pattern in the contralateral eye. Data were collected sitting during the diurnal/wake period and supine during the nocturnal/sleep period using a pneumatonometer (N = 30). Error bars, SD.

Fig 1 presents the overall 24-hour pattern in the CLS output signals. The pattern of 24-hour IOP in the contralateral eye is presented in Fig 2. The two 24-hour patterns had similarities. After the initiation of data collection, there was a continuous reduction in CLS output signals and a continuous IOP reduction toward the transition from the diurnal/wake period to the nocturnal/sleep period. The lowest CLS signal and the lowest IOP occurred near the end of the diurnal/wake period. Mean CLS signal and mean contralateral IOP value during the nocturnal/sleep period were higher than the related values during the diurnal/wake period. By visual inspection of Figs 1 and 2, the highest values of the two 24-hour data profiles occurred during the nocturnal/sleep period.

After 24-hour CLS wear, central corneal thickness decreased from 566.2 ± 33.5 μm to 554.9 ± 37.1 μm and the reduction of 11.3 ± 7.2 μm was significant (P< 0.001, paired t-test). There was no such change of central corneal thickness in the contralateral eye; 566.8 ± 31.2 μm at the beginning and 566.1 ± 34.9 μm at the end of the 24-hour period. There were differences in the cardiovascular parameters between the diurnal and nocturnal periods. Nocturnal mean blood pressure was 7.8% less than the diurnal mean blood pressure (6.7 ± 7.2 mmHg, P< 0.001) and nocturnal heart rate was 11.4% less than the diurnal heart rate (8.1 ± 4.7 beats/min, P< 0.001).

**Fig 3:**
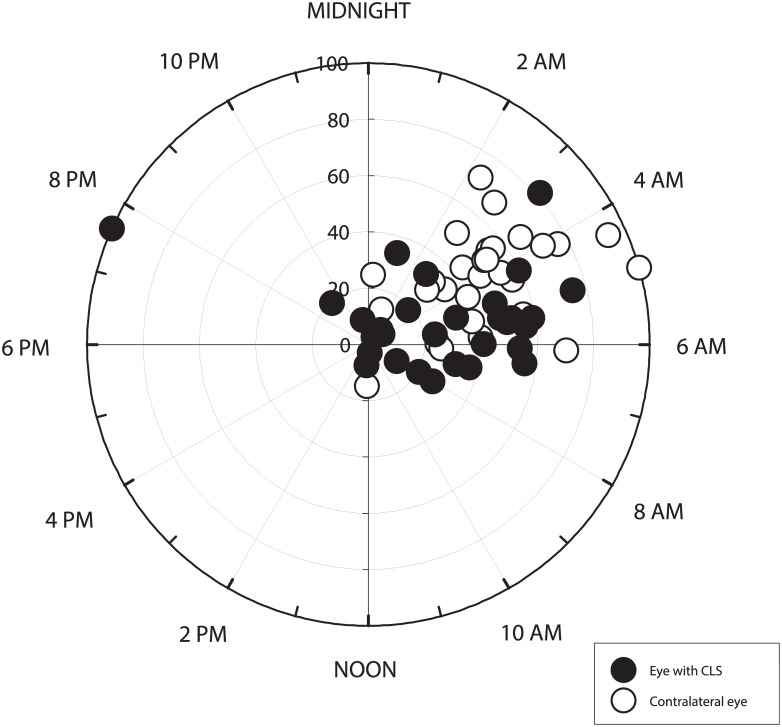
Estimated 24-hour rhythms in the paired eyes of healthy adults. Data were from contact lens sensor (CLS) output signals in one eye and measurements of intraocular pressure in the contralateral eye (N = 30). The clock time of the acrophase (phase timing) is shown with the amplitude in the radial scale as % of the maximal amplitude in the same group of eyes.

**Fig 4:**
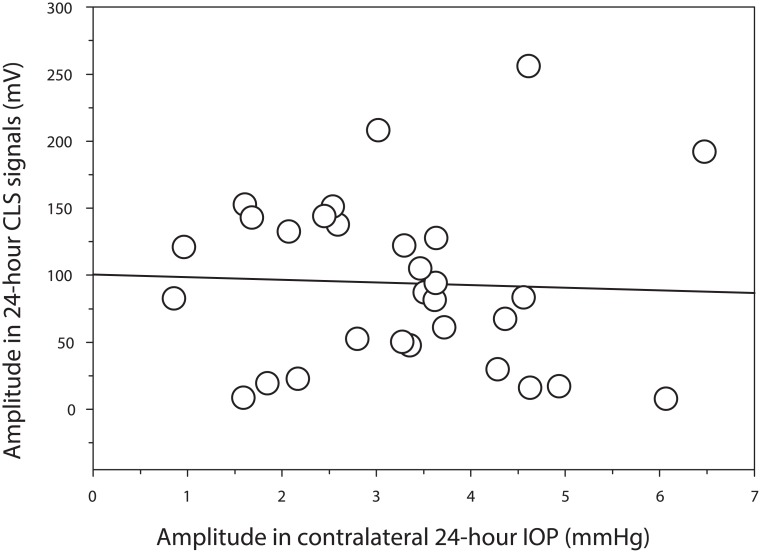
A lack of correlation between the simulated variations of 24-hour data in the paired eyes. The amplitude of simulated 24-hour rhythm using contact lens sensor (CLS) output signals and the amplitude of 24-hour intraocular pressure (IOP) using the pneumatonometer in the contralateral eye were analyzed (P = 0.820, linear regression, N = 30).

Using the technique of cosinor fitting, the acrophase (phase timing) and amplitude for the 24-hour CLS data as well as for the 24-hour IOP data in the contralateral eye were calculated. The Rayleigh test indicated that both the CLS output signals and the pneumatonometer data had a synchronized 24-hour phase timing. Fig 3 shows the distribution of acrophases and amplitudes for the two 24-hour rhythms. The peak timings for the 24-hour CLS data occurred at 4:44 AM ± 210 min and for the IOP data in the contralateral eye at 4:11 AM ± 120 min. The difference between the paired peak timings was not statistically significant (P = 0.256, Wilcoxon signed-rank test). The amplitude for the 24-hour CLS rhythm was 99.8 ± 65.5 mV and the amplitude for the 24-hour IOP rhythm was 3.1 ± 1.4 mmHg. Linear regression analysis showed no significant correlation between these two data variations (Fig 4; r = 0.043, P = 0.820).

Median of the absolute time interval between the paired peak timings (CLS/pneumatonometer) was 2 hour and 4 min (ranged from 4 min to 9 hour and 12 min) in the present group of 30 healthy individuals, which was significantly different (P<0.01, Mann-Whitney rank-sum test) from the median of 53 min (ranged from 2 min to 10 hour and 26 min) when bilateral IOP data were obtained using only the pneumatonometer from 91 healthy individuals [28]. For the latter group, there was a significant correlation between the simulated variations of 24-hour IOP data in the paired eyes (r = 0.774, P<0.001; linear regression analysis).

## Discussion

In this study, the estimated group mean of 24-hour peak timing obtained with the CLS was not statistically different from the estimated group mean of 24-hour IOP peak timing in the contralateral eye obtained with the pneumatonometer. As the reported group means of 24-hour IOP peak with pneumatonometry are outside usual office hours in previous studies [20]-[22], [24], [25], the CLS technology has the potential to estimate the group 24-hour peak timing. The CLS estimated that 77% (23/30) of simulated peak timings occurred during the nocturnal/sleep period for individuals housed in the sleep laboratory. For comparison, CLS recording at home in glaucoma patients showed that 66–74% of the peak timings appeared during the nocturnal/sleep period using the same cosinor fitting technique [27]. However, our results from this group of healthy adults showed a lack of correlation between the amplitudes of simulated 24-hour CLS output signals and 24-hour IOP in the contralateral eye. Since relatively symmetrical 24-hour rhythms including the parameters of peak timing and data variation were anticipated in the paired eyes of healthy individuals [28], this lack of correlation reflects the difficulty in the conversion of CLS output signals to accurate IOP values using a general formula. Estimated 24-hour IOP rhythms using the two devices should not be considered interchangeable.

The current CLS with its software configuration can provide up to 288 data points during a 24-hour period, which is more comprehensive than the 12 data points obtained in the sleep laboratory using a pneumatonometer. With more data points, an estimation of the 24-hour rhythm can be improved. However, as the wireless CLS technology is new, currently uncharacterized factors may cause more data variations. The uncharacterized factors may include the unknown relation between IOP and CLS output signals during the initial stage of wearing contact lens when the arbitrary reference unit was set by the manufacturer, the effect of unilateral CLS fitting on the sleep posture and IOP, and signal baseline drift [32], [33]. Since our calculations were based on the CLS raw data from all available 30 healthy subjects, these uncharacterized factors may contribute to the relatively large data variation in the simulated peak timings.

The CLS uses an embedded strain gauge that senses the circumferential curvature in the corneoscleral region [10], [11]. The relationship between IOP change and the volume change in the corneoscleral region has not been fully characterized. The pneumatonometer provides IOP values when the applanation state in the central cornea occurs between an externally applied pressure and IOP [34]. Since these two different techniques were used to obtain IOP-related data from the paired eyes, the absolute time difference in the estimated 24-hour peak timings was likely to be larger than the absolute time difference in the paired IOP peaks when only the pneumatonometer was used [28]. Judging the group medians, the absolute time difference associated with the use of two devices was increased moderately by 71 min. This magnitude of change may or may not be clinically significant; the increase was within the time interval of 2 hours scheduled for IOP measurements using the pneumatonometer. It is notable that data collected every 2 hours using the pneumatonometer may have missed some maximal and minimal values that would have been obtained with the CLS data recordings every 5 minutes.

With the advantage of collecting data continuously, the 24-hour CLS data profile may be used to evaluate the impact of awakening subjects during sleep for the IOP measurements. There were rhythmic data variations in the CLS output signals near the times of nocturnal IOP measurement at 1:30 AM, 3:30 AM, and 5:30 AM (see Fig 1). When the nocturnal IOP measurements were taken, the drop of mean blood pressure from the diurnal value was not in the commonly reported 10–20% dipper range for ambulatory blood pressure monitoring [6], [35]. Although the sample size is small, these observations suggest that some artifacts on blood pressure may have occurred due to awakening subjects for the nocturnal IOP measurements. However, the impact may not significantly affect the estimation of 24-hour peak timing as reported previously in another study [36].

There are unavoidable cornea-related issues when using the CLS in comparison with direct IOP monitoring using an intraocular wireless sensor [8], [37]. Our results showed a significant decrease of central corneal thickness of 11 μm in the CLS fitted eye at the end of 24-hour laboratory experiment. The cause is unclear. While endogenous swelling of central corneal thickness was expected during the nocturnal/sleep period for both eyes [38], [39], the CLS clinical trial associated with the present study did not examine the change in the central or peripheral corneal thickness during the nocturnal/sleep period [39], [40]. Whether or not corneal swelling at night may affect the sensing mechanism of CLS is unclear.

In summary, cosinor fitting of 24-hour data using the newly developed CLS presented a simulated peak timing that was not significantly different from the simulated 24-hour IOP peak timing in the contralateral eye using the pneumatonometer. More studies are needed to determine whether or not 24-hour CLS data is useful in the estimation of habitual 24-hour IOP peak timing. However, the current CLS technology does not provide a reliable estimate of 24-hour IOP variation.
